# A progressive increase in cardiovascular risk assessed by coronary angiography in non-diabetic patients at sub-diabetic glucose levels

**DOI:** 10.1186/1475-2840-10-56

**Published:** 2011-06-24

**Authors:** Sven Schinner, Reiner Füth, Kerstin Kempf, Stephan Martin, Holger S Willenberg, Matthias Schott, Wilfried Dinh, Werner A Scherbaum, Mark Lankisch

**Affiliations:** 1 Department of Endocrinology, Diabetes and Rheumatology, University Hospital Düsseldorf; Germany; 2 Heart Center, Helios Clinic Wuppertal, Wuppertal, Germany; 3 West-German Centre of Diabetes and Health, Düsseldorf Catholic Hospital Group, Düsseldorf, Germany; 4 Sana Hospital Gerresheim, Sana Clinics Düsseldorf GmbH, Düsseldorf, Germany

**Keywords:** impaired glucose tolerance, impaired fasting glucose, diabetes mellitus, oral glucose tolerance test, cardiovascular disease

## Abstract

**Objective:**

Diabetes mellitus type 2 (DM2) is a risk factor for coronary heart disease (CHD). While there is a clear correlation of fasting blood glucose (FBG) and 2 h post-challenge blood glucose values (2h-BG) with microvascular complications, the risk for CHD conferred by glucose dysregulation antecedent to DM2 is less clear. Therefore, we investigated associations of FBG and 2h-BG values with the prevalence of CHD assessed by coronary angiography as the most sensitive diagnostic tool.

**Research Design and Methods:**

Coronary angiography was performed in 1394 patients without known DM. Capillary blood glucose was analyzed before and 2 h after an oral glucose tolerance test. Associations between FBG as well as 2h-BG levels and the risk for CHD were assessed by logistic regression analysis.

**Results:**

1064 (75%) of patients were diagnosed with CHD. 204 (15%) were diagnosed with so far unknown DM2, 274 (20%) with isolated impaired fasting glucose (IFG), 188 (13%) with isolated impaired glucose tolerance (IGT) and 282 (20%) with both, IGT and IFG. We found a continuous increase in the risk for CHD with fasting and post-challenge blood glucose values even in the subdiabetic range. This correlation did however not suggest clear cut-off values. The increase in risk for CHD reached statistical significance at FBG levels of > 120 mg/dl (Odds Ratio of 2.7 [1.3-5.6] and 2h-BG levels > 140 mg/dl (141-160 mg/dl OR 1.8 [1.1-2.9], which was however lost after adjusting for age, sex and BMI.

**Conclusions:**

In our study population we found a continuous increased risk for CHD at fasting and 2h-BG levels in the sub-diabetic glucose range, but no clear cut-off values for cardiovascular risk.

## Introduction

Diabetes mellitus type 2 (DM2) is a major risk factor for micro- and macrovascular complications like coronary heart disease (CHD)[1,2]. While there is a clear correlation of fasting blood glucose (FBG) and 2 h post-challenge blood glucose values (2h-BG) with microvascular diseases, the risk for CHD conferred by glucose dysregulation antecedent to DM2 is less clear. There is controversial data concerning the correlation of blood glucose levels in the sub-diabetic range (impaired fasting glucose (IFG) and impaired glucose tolerance (IGT)) with the cardiovascular risk. The controversies might be due to different clinical end-points. Frequently used end-points are all-cause and cardiovascular mortality. Using all-cause mortality as an end-point Sorkin et al found a significant 40% increase in risk when the FBG exceeded 110 mg/dl [3]. This was in line with the Rancho Bernado study and the IPC Center study[4,5]. This increase in mortality further doubled within the FBG range between 126 mg/dl and 139 mg/dl[3]. There might also be gender differences as data from the Rancho Bernado study showed a significant increase in mortality within the FBG range between 100-110 mg/dl in men but not in women [6,7].

Consistently, a number of studies found a continuous increase in all-cause mortality with FBG levels in the sub-diabetic range although they did not reach statistical significance[8-11].

On the other hand, there are data from other studies reporting no correlation between FBG and mortality in the subdiabetic range[6].

Similarly, the relation between IGT and cardiovascular risk remains unclear. The ARIC study for example found no association of IGT with cardiovascular risk over a 6.3 year follow-up period[12]. However, the largest meta-analysis in that area including 20 prospective studies and almost 100 000 individuals revealed an increased cardiovascular risk for people with IGT when analysing clinical cardiovascular events[13].

In order to clarify the association of subdiabetic glucose values and cardiovascular risk we have chosen coronary angiography as a sensitive clinical end-point in the current study. Coronary atherosclerosis precedes the clinical manifestation of CHD and can be diagnosed early by coronary angiography. Patients undergoing coronary angiography show a high prevalence of undiagnosed glucose abnormalities[14]. There is only limited data on the correlation of glucose dysregulation with coronary atherosclerosis assessed by angiography. The only large cohort (n = 1040) has been reported by Saely and colleagues. They investigated the relation of impaired glucose tolerance (2h-BG levels in an oGTT) with angiographically characterised coronary atherosclerosis. They found IGT to be associated with an increased prevalence of coronary atherosclerosis but not with significant stenosis (defined as lumen narrowing > 50%)[15]. To our knowledge there is no data from a large cohort on angiographically assessed CHD in correlation to fasting blood glucose levels in the subdiabetic range. Therefore, the aim of this study was to investigate if the prevalence of coronary stenosis assessed by coronary angiography is increased at subdiabetic glucose levels.

## Methods

### Study population

All patients (n = 1394) without known DM2 that have been submitted to the heart center Wuppertal (Germany) for elective coronary angiography between 2007 and 2009 were enrolled in this retrospective study. The study was performed according to the rules of the Declaration of Helsinki and all study patients gave informed written consent. The clinical and demographic data obtained for each patient included sex, age, body mass index (BMI), history of CHD and actual cardiovascular intervention.

### Determination of coronary and glycaemic state

In each patient, coronary angiography was performed at least one day before the oGTT. Coronary heart disesase (CHD) was diagnosed in the presence of a luminal narrowing ≥50% of any epicardial vessel. An oGTT with 75 g (DextroOGT, Roche Diagnostics, Mannheim, Germany) was performed and capillary blood glucose was measured before and 2 h after oral glucose load with a point-of-care system (ecoSolo II; CAREdiagnostica), which is based on enzymatic, amperometric measurement using a GOD-H_2_0_2 _electrode. Coefficient of variation was about 5%. Of note, classifications of glycaemic state were made according to the criteria of the German Diabetes Association (Deutsche Diabetes Gesellschaft) for capillary blood glucose (IFG > 90 mg/dl; IGT > 140 mg/dl; DM2 ≥ 110 mg/dl (fasting) or ≥ 200 mg/dl (2h-BG after oGTT)). For one patient the FBG value was missing. 2h-BG values were missing for 20 patients since there FBG values had been considerably higher than 110 mg/dl. Therefore, OGTT had not been performed in those cases and DM2 had been diagnosed based on the FBG values.

### Statistical analysis

Logistic regression analysis with adjustment to sex and age was used to determine the influence of FBG or 2h-BG on CHD risk. Associations were determined by Spearman correlation. The level of significance was 0.05. For data analysis GraphPad Prism 4.0 (GraphPad Software, San Diego, CA, USA) and SAS statistical package version 8.2 TS2MO (SAS Institute, Cary, NC, USA) were used.

## Results

### High prevalence of coronary heart disease in the study population

In this study we enrolled 1,394 patients without known DM2 who underwent elective coronary angiography. The patient characteristics are given in Table 1. As shown here, 76% of the patients were diagnosed with CHD based on the lumen narrowing ≥50% diagnosed by coronary angiography. The prevalence of CHD correlated with age (r = 0.12, p < 0.0001), male sex (r = 0.23; p < 0.0001), the diagnosis of DM2 (r = 0.08; p = 0.003), FBG (r = 0.07; p = 0.009), and 2H-BG (r = 0.14; p < 0.0001). Using an oGTT 15% of the patients were identified with so far unknown DM2, 20% with isolated IFG, 13% with isolated IGT and 20% with both IGT and IFG. Only 32% were normoglycaemic (Table 1).

**Table 1:** Patient characteristics

	Patients (n = 1394)
Sex [n] (male/female)	962 (69%)/432 (31%)

Age [years]	64.0 ± 11.4

Body Mass Index [kg/m^2^]	27.6 ± 4.5

CHD [n]	1064 (76%)

Acute myocardial infarction [n]	294 (21%)

CHD before [n]	579 (42%)

FBG [mg/dl]	92.5 ± 16.2

2h-BG [mg/dl]	145.0 ± 43.1

NGT [n]	446 (32%)

Isolated IFG [n]	274 (20%)

Isolated IGT [n]	188 (13%)

IFG/IGT [n]	282 (20%)

DM2 [n]	204 (15%)

CHD, coronary heart disease; FBG, fasting blood glucose; 2h-BG, blood glucose 2 h after 75 g oral glucose challenge; NGT, normal glucose tolerance; IFG, impaired fasting glucose; IGT, impaired glucose tolerance; DM, diabetes mellitus

### Increased risk for coronary heart disease with blood glucose levels in the subdiabetic range

**Figure 1:**
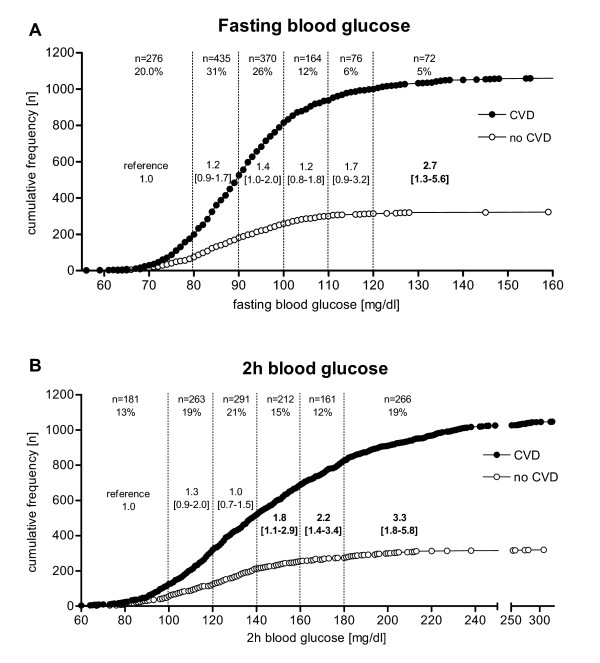
**Cumulative frequency of fasting and 2 h blood glucose values for patients with and without coronary heart disease**. Cumulative frequencies of patients with and without coronary heart disease (CHD) were plotted against blood glucose values in (**A**) fasting state and (**B**) 2 h after an oral glucose tolerance test. Blood glucose ranges were divided into sextiles with the lowest sextile defined as reference group each. Shown are the numbers and percentages of patients as well as the odds ratios for CHD in the respective blood glucose sextiles.

Capillary blood glucose was determined in the fasting state and 2 h after an oGTT. The distribution of FBG levels among the patients was as followed: 435 (31%) were < 90 mg/dl (329 with CHD vs. 106 without CHD), 370 (26%) were in the range of 91-100 mg/dl (292 vs. 78), 164 (12%) were between 101-110 mg/dl (124 vs. 40), 76 (6%) were between 111-120 mg/dl (62 vs. 14) and 72 (5%) were > 120 mg/dl (63 vs. 9) (Figure 1A). Patients with FBG ≤80 mg/dl (n = 276 (20%); 200 vs. 76) were defined as the reference group. Compared to the reference group the risk for CHD increased continuously with FBG levels. Table 2 shows that statistical significance was reached at FBG levels > 120 mg/dl (OR 2.67 [1.3-5.6]) and remained significant after adjusting for sex and age but not in a separate model including the BMI. As seen in Table 3 when blood glucose was expressed as a continuous variable, both the fasting as well as the 2 h glucose values were significantly correlated with CHD risk.

**Table 2:** Association between blood glucose levels and coronary heart disease.

fasting blood glucose [mg/dl]	rohes OR [CI]	adj. OR [CI]^1^	adj. OR [CI]^2^
≤ 80 (reference)	1.00	1.00	1.00

81-90	1.18 [0.88-1.66]	1.13 [0.79-1.64]	1.15 [0.78-1.71]

91-100	1.42 [0.99-2.04]	1.24 [0.85-1.81]	1.27 [0.84-1.92]

101-110	1.17 [0.75-1.83]	1.04 [0.65-1.67]	1.12 [0.67-1.87]

111-120	1.69 [0.89-3.20]	1.39 [0.71-2.74]	1.41 [0.67-2.97]

> 120	**2.67 [1.27-5.64]**	**2.50 [1.15-5.46]**	2.27 [0.93-5.31]

**2 h blood glucose [mg/dl]**			

≤ 100 (reference)	1.00	1.00	1.00

101-120	1.30 [0.86-1.98]	1.17 [0.74-1.82]	0.99 [0.61-1.60]

121-140	0.99 [0.66-1.47]	0.87 [0.56-1.33]	0.74 [0.46-1.17]

141-160	**1.80 [1.14-2.86]**	1.45 [0.89-2.38]	1.48 [0.86-2.53]

161-180	**2.17 [1.40-3.37]**	**2.18 [1.18-4.06]**	1.76 [0.90-3.45]

> 180	**3.25 [1.83-5.78]**	1.53 [0.92-2.53]	1.44 [0.80-2.57]

^1 ^adjusted for sex and age

^2 ^adjusted for sex, age and bmi

**Table 3:** Associations between blood glucose levels/glucose tolerance state and coronary heart disease

	raw OR [CI]	adj. OR [CI]^1^	adj. OR [CI]^2^
fasting blood glucose	1.013 [1.004-1.022]	1.010 [1.001-1.019]	1.010 [1.000-1.020]

2 h blood glucose	1.008 [1.005-1.011]	1.007 [1.003-1.010]	1.007 [1.003-1.011]

NGT/IFG+IGT/DM2 combined	1.571 [1.292-1.910]	1.401 [1.147-1.727]	1.446 [1.158-1.807]

^1 ^adjusted for sex and age

^2 ^adjusted for sex, age and B.M.I.

Consistently, Table 4 shows a continuous risk increment for CHD over the whole range of glucose levels.

**Table 4:** Difference in numbers of patients with or without coronary heart disease at defined blood glucose levels

fasting blood glucose [mg/dl]	p
≤ 80 vs. > 80	0.067

≤ 90 vs. > 90	**0.031**

≤ 100 vs. > 100	0.171

≤ 110 vs. > 110	**0.018**

≤ 120 vs. > 120	**0.031**

**2 h blood glucose [mg/dl]**	

≤ 80 vs. > 80	0.153

≤ 90 vs. > 90	**0.001**

≤ 100 vs. > 100	**0.011**

≤ 100 vs. > 100	**0.009**

≤ 100 vs. > 100	**< 0.0001**

≤ 100 vs. > 100	**< 0.0001**

≤ 100 vs. > 100	**0.006**

When analysing post-challenge glucose, we found 181 (13%) of the patients had 2h-BG values ranging 101-120 mg/dl (196 vs. 67), 291 (21%) were in the range of 121-140 mg/dl (200 vs. 91), 212 (15%) in the range of 141-160 mg/dl (170 vs. 42), 161 (12%) were between 161-180 mg/dl (142 vs. 19), and 266 (19%) were > 180 mg/dl (221 vs. 45) (Figure 1B). Patients with 2h-BG levels ≤100 mg/dl (n = 181 (13%); 125 vs. 56) were defined as the reference group. Similarly to our findings for fasting glucose the CHD risk increased at subdiabetic 2 h glucose levels with an increased risk for CHD with an OR 1.80 [1.1-2.9] for patients within the glucose range of 141-160 mg/dl, 2.2 [1.4-3.4] for those within 161-180 mg/dl and 3.3 [1.8-5.8] for patients whose 2h-BG exceeded 180 mg/dl (Table 2).

## Discussion

In our study population 76% of patients were diagnosed with CHD and 15% with so far unknown DM2, 20% with IFG, 13% with IGT and 20% with both, IFG and IGT. DM2 is a major risk factor for coronary heart disease[16]. However, the diagnostic thresholds to define diabetes are based on microvascular endpoints. Therefore, it is not clear whether microvascular and CHD risk increases at the same glucose thresholds. In our study, the risk for CHD as assessed by coronary angiography increased with the blood glucose levels even in the subdiabetic range. However, we found a rather continuous increase of CHD risk with blood glucose. In the *fasting state *the increase in risk reached statistical significance only when the capillary fasting glucose levels were > 120 mg/dl. It is important to note that the diabetes threshold in the fasting state in capillary blood is ≥ 110 mg/dl. Therefore, in our study IFG increased the risk for CHD but did not reach statistical significance. When analysing *post-challenge glucose *values we found a significant increase in CHD risk even at the level of IGT. The analysis of the combination of all grades of glucose disturbancies (IFG, IGT and diabetes) revealed a positive and significant correlation with CHD (table 3).

There is ongoing debate if there is at all a clear threshold for CHD risk or rather a continuous increase with increasing blood glucose levels. Therefore, the most important finding in our study is the increase in the prevalence of coronary heart disease with blood glucose levels in the subdiabetic range. Our results are in line with a large retrospective meta-analysis including 95,000 patients demonstrating an increase for cardiovascular events even at sub-diabetic glucose levels[13]. In addition, a recent meta-analysis identified a positive correlation between IGT and carotid intima media thickness as a marker for cardiovascular risk[17]. In line with this study we found that postprandial hyperglycaemia contributes more to CHD than fasting hyperglycaemia. However, various investigations on this question yielded controversial data. For example in the prospective ARIC (Atherosclerosis Risk in Communities) study there was no association of isolated IFG or IGT with cardiovascular risk. In line with this, a recent study by Pereg and colleagues attributed the progressive increase in cardiovascular risk at fasting glucose levels in the upper normal range to the prevalence of comorbidities rather than to glucose effects[18].

Notably, the endpoints in this study - as in most other similar studies - were clinical events. This included all cause mortality, clinically incident CHD and surrogate parameters for cardiovascular risk e.g. intima-media thickness, ankle-brachial blood pressure index and left ventricular hypertrophy.

However, coronary angiography is the current diagnostic gold standard for early detection of coronary artery disease[19]. Therefore, we investigated the correlation of blood glucose levels with data from patients undergoing elective coronary angiography.

Although patients with known DM2 had not been included in the study, two thirds of the patients had either IFG, IGT, or newly DM2 and only 32% of the patients included turned out to be normoglycaemic. This is in line with previous data[14,20] and furthermore emphasises the importance of diagnostic efforts to detect DM2 in patients suspicious for CHD. How reliable are these oGTT data gained in hospital? A recent study reported follow-up data from oGTT tests done in hospital and after three months in patients with ST-elevation myocardial infarction. They found a large part of the study population to change the metabolic state with time[21]. However, it is not known, whether the patients were still critically ill during the in hospital oGTT. In our current study we had a different situation as we excluded patients with acute myocardial infarction.

There is very limited data on cardiovascular atherosclerosis assessed by angiography with respect to sub-diabetic glucose levels. Earlier studies including relatively small numbers of patients that investigated the correlation of IGT with coronary artery disease by angiography demonstrated controversial results[22-25]. Only one large study by Saely and colleagues investigated coronary atherosclerosis by angiography with respect to IGT and found a positive correlation. In addition to this, our study correlates the risk for CHD defined as significant lumen narrowing with IGT and with distinct levels of FBG in the sub-diabetic range. We confirm the significant increase in CHD with IGT. In addition, there is the same trend of a continuous increase in CHD risk for fasting glucose values in the subdiabetic range; the effect was however significant only above the diabetes threshold. To our knowledge, this is the first study investigating this correlation in a large cohort of patients undergoing angiography. One hypothesis to explain this finding is that post-challenge glucose excursions contribute a greater CHD risk than slightly elevated fasting levels.

Our study has several limitations: First, it is a retrospective study, therefore not directly allowing the prediction of future cardiovascular risk in patients at a given sub-diabetic glucose level. Second, we did not have the option to concisely include effects of co-medication (e.g. acetylsalicylic acid, statins) in our analysis. Third, we did not have a chance to evaluate the extent of coronary atherosclerosis. Instead, we defined the diagnosis of significant coronary stenosis when lumen narrowing was ≥50%. However, based on the high number of patients included (n = 1394) we are confident that the effects of blood glucose on coronary atherosclerosis observed in our study are robust and reliable to assess the risk for coronary heart disease in the sub-diabetic glucose range. Interestingly, Saely et al. found a significant increase in the prevalence of coronary atherosclerosis (defined as any visible lumen narrowing) in patients with IGT but this was not significant for relevant stenosis (defined as lumen narrowing > 50%). On the other hand, they found 2h-BG as a continuous variable to be significantly correlated with both prevalence of atherosclerosis and relevant stenosis[19]. In addition, a recent follow-up study on this population over 3.8 years showed a positive correlation of IFG with future cardiovascular events[26]. In line with this, we found not only 2h-BG but also FBG to be continuously correlated with increased prevalence of significant coronary stenosis. This seems to be in contrast to the effects of blood glucose on microvascular endpoints which show a steep increase of risk at distinct blood glucose values. Apparently, the risk of cardiovascular complications conferred by glucose is more a continuous gradual increase. This is important in identifying people at risk for cardiovascular disease in populations that do not fulfil the current diagnostic criteria of DM2. Taken together; our study demonstrates a continuous increase in cardiovascular risk at sub-diabetic glucose levels in patients undergoing elective coronary angiography.

## Conflict of interests

The authors declare that they have no competing interests.

## Authors' contributions

WAS, MS, SM made substantial contribution to conception and design. KK, SS, RF, WD and ML made substantial contributions to acquisition of data, or analysis and interpretation of data. HW has been involved in drafting the manuscript or revising it critically for important intellectual content. All authors read and approved the final manuscript.
